# Supplementary material for: MetaRibo-Seq measures translation in microbiomes
Source: Nat Commun. 2020 Jun 29;11:3268. doi: 10.1038/s41467-020-17081-z (PMC7324362; doi:10.1038/s41467-020-17081-z)
Supplement: Supplementary file 10 — Supplementary Data 7 [file 41467_2020_17081_MOESM10_ESM.zip › File2/Confidence_High_Taxonomy/386457_out.krona.html]

Javascript must be enabled to view this page.

members
magnitude
magnitudeUnassigned
count
unassigned
taxon
rank

386457\_out

82

superkingdom
2
82

phylum
976
82

82
200643
class

order
82
171549

171550
2
family

2
239759
genus


SRS148784\_contig\_number\_15835
species
1896969
1


SRS019178\_contig\_number\_4620
species
328814
1

815
80
family

80
816
genus


SRS147557\_contig\_number\_15450
species
1
457392

species

SRS077730\_contig\_number\_11597
1
246787

1
46506

SRS077589\_contig\_number\_1062
species

1
1946083
species

SRS1041145\_contig\_number\_4342


SRS015960\_contig\_number\_6468
species
28116
1

species

SRS019787\_contig\_number\_2523
151276
1

species

SRS1055099\_contig\_number\_4321
1
28111

species

SRS011084\_contig\_number\_14055SRS012902\_contig\_number\_317SRS013158\_contig\_number\_2756SRS014736\_contig\_number\_7873SRS015190\_contig\_number\_8665SRS015264\_contig\_number\_21398SRS015578\_contig\_number\_5745SRS016132\_contig\_number\_2103SRS016267\_contig\_number\_576SRS017433\_contig\_number\_14149SRS017521\_contig\_number\_19339SRS018313\_contig\_number\_9657SRS018541\_contig\_number\_9058SRS018817\_contig\_number\_16546SRS018888\_contig\_number\_665SRS019267\_contig\_number\_5892SRS019397\_contig\_number\_10296SRS020869\_contig\_number\_32081SRS023346\_contig\_number\_182SRS023829\_contig\_number\_300SRS023971\_contig\_number\_1780SRS024075\_contig\_number\_743SRS024388\_contig\_number\_8160SRS024549\_contig\_number\_1897SRS043411\_contig\_number\_11464SRS043701\_contig\_number\_10490SRS046712\_contig\_number\_276SRS049402\_contig\_number\_10096SRS050299\_contig\_number\_9277SRS050941\_contig\_number\_21999SRS051610\_contig\_number\_2840SRS053214\_contig\_number\_2308SRS053573\_contig\_number\_1207SRS055966\_contig\_number\_236SRS057717\_contig\_number\_1034SRS058723\_contig\_number\_6500SRS063127\_contig\_number\_491SRS063985\_contig\_number\_16350SRS075398\_contig\_number\_928SRS075878\_contig\_number\_848SRS076804\_contig\_number\_12466SRS077194\_contig\_number\_10720SRS077335\_contig\_number\_1292SRS078419\_contig\_number\_35520SRS097920\_contig\_number\_18011SRS098655\_contig\_number\_387SRS098655\_contig\_number\_571SRS098881\_contig\_number\_13402SRS104036\_contig\_number\_6393SRS1041129\_contig\_number\_3378SRS104311\_contig\_number\_19482SRS104485\_contig\_number\_830SRS104636\_contig\_number\_16493SRS1055043\_contig\_number\_1087SRS140492\_contig\_number\_26836SRS140513\_contig\_number\_9032SRS142599\_contig\_number\_14850SRS143342\_contig\_number\_9875SRS143417\_contig\_number\_18684SRS143598\_contig\_number\_13057SRS144362\_contig\_number\_11627SRS144506\_contig\_number\_8853SRS146812\_contig\_number\_16206SRS147022\_contig\_number\_1782SRS147272\_contig\_number\_424SRS147377\_contig\_number\_9526SRS147653\_contig\_number\_983SRS148091\_contig\_number\_1045SRS148159\_contig\_number\_43583SRS893279\_contig\_number\_11198SRS893342\_contig\_number\_1429
817
71


SRS075021\_contig\_number\_426
species
357276
1

329854
1

SRS056259\_contig\_number\_11312
species
